# Optimizing Lumefantrine Dosing for Young Children in High-Malaria-Burden Countries Using Pharmacokinetic-Pharmacodynamic Simulations

**DOI:** 10.1093/ofid/ofae627

**Published:** 2024-10-17

**Authors:** Segolene Simeon, Emma Hughes, Erika Wallender, Belén P. Solans, Rada Savic

**Affiliations:** Department of Bioengineering and Therapeutic Sciences, University of California, San Francisco, San Francisco, California, USA; Department of Bioengineering and Therapeutic Sciences, University of California, San Francisco, San Francisco, California, USA; Department of Clinical Pharmacy, University of California, San Francisco, San Francisco, California, USA; Department of Bioengineering and Therapeutic Sciences, University of California, San Francisco, San Francisco, California, USA; Department of Bioengineering and Therapeutic Sciences, University of California, San Francisco, San Francisco, California, USA

**Keywords:** COVID-19, Omicron, post-COVID symptoms, SARS-CoV-2

## Abstract

**Background:**

Artemether-lumefantrine is the most widely used treatment for uncomplicated malaria and it is dosed based on weight bands according to World Health Organization (WHO) guidelines. However, children are vulnerable to underdosing. Inadequate dosing can lead to treatment failure and drug resistance.

**Methods:**

Nutritional parameters for 372 363 children <5 years old in 25 high-malaria-burden countries were acquired from the Demographic and Health Surveys program. Prevalence of attaining day 7 lumefantrine concentrations ≥200 ng/mL and remaining reinfection free for 42 days were evaluated using a simulation-based approach with a population pharmacokinetic-pharmacodynamic model. Besides the WHO-recommended lumefantrine dosing regimen (twice daily for 3 days), we explored 3 adjusted regimens: extended (2 extra days of dosing), increased (1 extra 120-mg tablet per dose), and intensified (thrice daily for 3 days). We also explored an alternative method dosing malnourished children based on expected weight for age.

**Results:**

We estimated that 75% of children reached the 200 ng/mL lumefantrine threshold and 77% were malaria free for 42 days when using WHO treatment guidelines. By switching to the alternative dosing method, 5% more children achieved target lumefantrine levels; 22% more achieved the target using the alternative dosing and the extended regimen. With combined alternative plus extended dosing, 97% of children reached 200 ng/mL lumefantrine and 88% were malaria free for 42 days.

**Conclusions:**

This study highlights the inadequacies of weight-based lumefantrine dosing for young and underweight children and supports the need of clinical trials using extended dosing based on expected weight in malnourished children.

Malaria is transmitted through the bite of female *Anopheles* mosquitoes infected with the *Plasmodium* parasite. In 2022, 249 million cases of malaria and 608 000 deaths were estimated worldwide, with 76% of deaths occurring in children <5 years of age [1]. An essential tool in reducing childhood malaria morbidity and mortality is to provide an effective malaria treatment to avoid parasite recrudescence and reinfection. Doing so, the transmission rate can be reduced and drug resistance limited [2]. In combination with prevention strategies, such as the use of nets, the World Health Organization (WHO) aims to eliminate malaria infection across the globe [3].

Artemisinin-based combination therapies are highly effective first-line malaria treatments. Artemether-lumefantrine is the most widely used and the current WHO guidelines, which recommend weight-based dosing regimens for children, were extrapolated from adult clinical studies [3]. However, physiological differences can alter the pharmacokinetic (PK) and pharmacodynamic (PD) properties of drugs, and using weight alone as the dosing reference may result in underdosing malnourished and young children, potentially impacting safety and efficacy [4]. For drugs such as piperaquine, additional efficacy studies have shown that the initial recommended dosing was not adequate because of lower drug exposure in young children. To improve drug exposure, the dose has been adjusted by reducing the weight-band range size [3, 5, 6]. Lumefantrine (LF) exposure is crucial to prevent malaria recrudescence, and the day 7 concentration is a significant factor in determining therapeutic response [7, 8]. Some studies showed that to control treatment failure, LF exposure was the most suitable determinant over clinical and molecular factors [9]. Furthermore, the LF concentration cutoff of 200 ng/mL at day 7 is commonly used as an accurate threshold to prevent malaria recrudescence [10–12]. Most countries with a high malaria burden are also affected by a high malnutrition prevalence. Every year, approximately 3.1 million children under 5 die because of malnutrition, which represents 45% of childhood deaths [13]. The complex relationship between malaria and malnutrition is recognized, but its understanding is poor and needs to be deepened. It has been shown that malaria worsens malnutrition, and chronic malnutrition is a risk factor for malaria infection as it may reduce antimalarial immunity and drug exposure due to insufficient dosing and decreased absorption and/or bioavailability [14]. In addition, patients with a lower immunity to *Plasmodium falciparum* may have lower favorable treatment responses or may need higher drug concentrations. Some studies showed that LF concentrations on day 7 were lower in underweight children aged <3 years compared with well-nourished children or adults [11, 12, 15].

The relatively small studies that have detected PK differences by nutrition status have not been powered to detect the clinical consequences of this decreased LF exposure on malaria treatment outcomes, and WHO still recommends using weight-band dosing [3]. Furthermore, we would need to collect large region-specific weight-for-age data to guide expected weight-based dosing, which can be challenging [16].

As a support of the Chotsiri et al study [12], this study aims to quantify the impact of malnutrition on LF exposure and the risk of recrudescent malaria in young children, using a large database of nationally representative health and nutrition data from the Demographic and Health Surveys (DHS) program in 25 countries with high prevalence of malaria and malnutrition [17]. Using a simulation-based approach with a population PK-PD model, we compared the LF exposure with the recommended WHO weight-based LF dosing regimen, as well as feasible optimized and adjusted dosing regimens for young children at high risk for malaria with and without malnutrition.

## METHODS

### Data Sources

A database of anthropometric and nutritional measures in children <5 years old was created using survey data from the DHS program [17] and Chotsiri et al [12]. DHS data from the 25 countries with the highest malaria-attributable mortality were included [13, 14] (Table 1). The database included age, sex, weight, height, and indicators related to malnutrition status and severity (height-for-age *z*-score [HAZ], weight-for-age *z*-score [WAZ], and weight-for-height *z*-score [WHZ]) for children 3–59 months old. The mid-upper arm circumference (MUAC) was imputed for every child according to Chotsiri and colleagues’ data [12]. Children <3 months old were excluded, since maternal antibodies are still present and provide protection, making the prevalence of malaria in this population very low. The measures of malnutrition are expressed according to standard deviation with respect to a reference mean value. Underweight, stunted, and wasted nutritional conditions are respectively defined as WAZ < −2, HAZ < −2, and WHZ < −2 [14, 18]. In addition to a child's actual weight, their expected weight was calculated based upon their age in months and sex using the WHO's Child Growth Standard Charts [19] corresponding to the weight for their age if WAZ = 0. For the Central African Republic, *z*-score data were not available, and the WHO Anthro R Package was used to calculate them.

**Table 1.** Characteristics of the Demographic and Health Surveys Child Population

Country	No. of Children	Underweight^a^, No. (%)	Stunted^a^, No. (%)	Wasted^a^, No. (%)	Age^b^, mo	Weight^c^, kg	Age-Based Weight^d^, kg	WAZ^c^	HAZ^c^	WHZ^c^
All countries	372 363	105 376 (28)	140 228 (38)	54 917 (15)	30	10.9 (2.5, 34)	13.1	−1.32 (−5.9, 5)	−1.56 (−6, 6)	−0.64 (−5.6, 5.9)
Angola	5916	1130 (19)	2275 (38)	297 (5)	29	11.2 (3.6, 27)	12.7	−1.02 (−5.2, 4.5)	−1.62 (−5.9, 5.9)	−0.16 (−4.8, 4.8)
Benin	10 921	1837 (17)	3574 (33)	551 (5)	29	11.2 (3.1, 24)	12.7	−1.03 (−5.6, 3.6)	−1.47 (−6, 5.9)	−0.28 (−4.9, 4.9)
Burkina Faso	6229	1585 (25)	2205 (35)	940 (15)	29	10.8 (3.1, 26)	12.9	−1.23 (−5.3, 3.7)	−1.48 (−6, 5.6)	−0.58 (−4.9, 5)
Burundi	5751	1700 (30)	3225 (56)	290 (5)	30	10.8 (3.8, 23)	12.9	−1.44 (−5.4, 4)	−2.18 (−6, 4.6)	−0.28 (−4.8, 5)
Cameroon	4199	425 (10)	1216 (29)	162 (4)	29	12.3 (3.9, 28)	12.9	−0.33 (−5.7, 4.3)	−1.14 (−6, 6)	0.43 (−5, 5)
Central African Republic	2159	512 (24)	905 (42)	207 (10)	17	9.1 (3.7, 18)	10.5	−1.08 (−5.6, 3.9)	−1.69 (−6, 5.9)	−0.32 (−5.6, 5.9)
Chad	9355	3144 (34)	4179 (45)	1310 (14)	31	10.7 (3.4, 29)	13.3	−1.43 (−5.8, 4.7)	−1.77 (−6, 5.9)	−0.61 (−4.9, 4.8)
Cote d’Ivoire	3014	445 (15)	915 (30)	206 (7)	28	11.2 (2.9, 23)	12.7	−0.83 (−5.3, 3.4)	−1.34 (−6, 5.9)	−0.16 (−4.7, 4.9)
DRC	7662	1833 (24)	3493 (46)	584 (8)	29	10.9 (3.5, 26)	12.9	−1.13 (−5.3, 4.3)	−1.81 (−6, 5.9)	−0.17 (−4.9, 4.9)
Ethiopia	8363	2149 (26)	3136 (37)	998 (12)	29	10.9 (3.3, 30)	12.9	−1.24 (−5.8, 3.9)	−1.52 (−6, 6)	−0.58 (−4.9, 4.8)
Ghana	2598	294 (11)	514 (20)	122 (5)	29	11.4 (2.9, 22)	12.7	−0.78 (−5.7, 3.8)	−1.03 (−5.9, 5)	−0.28 (−4.7, 4.1)
Guinea	3190	506 (16)	1002 (31)	269 (8)	29	11.5 (4, 24)	12.9	−0.86 (−5.5, 3.6)	−1.24 (−6, 6)	−0.2 (−4.9, 4.7)
India	217 477	75 858 (35)	84 828 (39)	43 544 (20)	31	10.8 (2.5, 34)	13.3	−1.56 (−5.9, 4.9)	−1.61 (−6, 6)	−0.94 (−5, 5)
Kenya	17 890	2430 (14)	4952 (28)	980 (5)	30	11.6 (3.2, 29)	13.1	−0.80 (−5.4, 4.4)	−1.25 (−6, 6)	−0.14 (−5, 5)
Malawi	4880	579 (12)	1760 (36)	146 (3)	30	11.65 (3.5, 22)	13.1	−0.85 (−4.8, 4.4)	−1.61 (−6, 6)	0.05 (−4.9, 4.9)
Mali	7766	1473 (19)	2119 (27)	728 (9)	29	11.2 (3.4, 25)	12.7	−1.02 (−5.7, 3.8)	−1.17 (−6, 6)	−0.54 (−5, 5)
Mozambique	8895	1179 (13)	3565 (40)	446 (5)	28	11.5 (3.3, 24)	12.7	−0.75 (−5.4, 4.6)	−1.69 (−6, 5.8)	0.25 (−4.9, 5)
Niger	4530	1664 (37)	1965 (43)	836 (18)	30	10.4 (2.8, 25)	12.9	−1.58 (−5.7, 3.3)	−1.77 (−6, 5.8)	−0.86 (−5, 4.9)
Nigeria	10 772	2333 (22)	3992 (37)	721 (7)	29	11.2 (2.9, 25)	12.9	−1.05 (−5.6, 3.6)	−1.50 (−6, 6)	−0.27 (−5, 4.6)
Papua New Guinea	3077	617 (20)	1224 (40)	299 (10)	31	11.4 (3, 29)	13.3	−1.08 (−5.4, 4.9)	−1.65 (−6, 5.7)	−0.26 (−4.8, 5)
Sierra Leone	3856	550 (14)	1193 (31)	219 (6)	28	11.3 (3.2, 27)	12.5	−0.84 (−5.6, 4.5)	−1.35 (−6, 5.7)	−0.12 (−4.9, 4.8)
Tanzania	8420	1169 (14)	2931 (35)	374 (4)	28	11.3 (3, 25)	12.7	−0.91 (−5.6, 3.7)	−1.53 (−6, 6)	−0.07 (−5, 5)
Togo	3070	520 (17)	887 (29)	218 (7)	29	11.3 (3.4, 24)	12.9	−0.95 (−5.5, 4.7)	−1.27 (−6, 5.1)	−0.29 (−4.7, 4.6)
Uganda	4150	447 (11)	1224 (29)	144 (3)	29	11.8 (3.1, 24)	12.7	−0.66 (−5.1, 3.6)	−1.33 (−6, 5)	0.1 (−5, 5)
Zambia	8223	997 (12)	2949 (36)	326 (4)	29	11.6 (4, 24)	12.9	−0.8 (−5.4, 4.5)	−1.57 (−6, 6)	0.07 (−5, 5)

Abbreviations: DRC, Democratic Republic of the Congo; HAZ, height-for-age *z*-score; WAZ, weight-for-age *z*-score; WHZ, weight-for-height *z*-score.

^a^Number of Demographic and Health Surveys children with nutrition indicators related to undernutrition (underweight, WAZ < −2; stunted, HAZ < −2; wasted, WHZ < −2).

^b^Values are reported as median. Every country has a minimum, maximum age of 3, 59 months, except Central African Republic, which has a minimum, maximum age of 3, 35 months.

^c^Values are reported as median (minimum, maximum).

^d^The expected body weight is determined according to the age-based World Health Organization child's growth standard. Values are reported as median. Every country has a minimum, maximum age-based weight of 5.8, 18 kg, except Central African Republic, which has a minimum, maximum age-based weight of 5.8, 14 kg.

### Dosing Regimens

We focused on LF dosing optimization for this analysis, since early treatment failures attributed to artemisinin remain rare in high-burden countries and differences in the risk of treatment failure with LF have been associated with exposure to the longer-acting LF [20]. Table 2 summarizes the WHO dosing guidelines for lumefantrine as well as the alternative regimens explored. In addition to the current WHO-recommended LF regimen (standard: twice-daily dosing for 3 days), we explored 3 adjusted regimens: extended (2 extra days of twice-daily dosing), increased (1 extra tablet added per dose), and intensified (thrice-daily dosing for 3 days). In addition to these modifications, we explored an alternative dosing method in which children with good nutritional status (WAZ ≥ 0) received the WHO-recommended dosing by weight and children with poor nutritional status (WAZ < 0) received the WHO-recommended dose for their expected weight. With this method, malnourished children could receive higher doses than they would per WHO recommendations. We refer to the WHO-recommended dosing method using actual body weight only as the “WHO method” and the alternative method as “ALT method.” Furthermore, to simplify dose selection using the ALT method, we proposed dosing charts for girls and boys (Supplementary Figure 1).

**Table 2.** Lumefantrine Dosing Regimens Tested

Regimen	<15 kg	15–25 kg	25–35 kg
Standard	120 mg BID/3 d	240 mg BID/3 d	360 mg BID/3 d
Extended	120 mg BID/5 d	240 mg BID/5 d	360 mg BID/5 d
Increased	240 mg BID/3 d	360 mg BID/3 d	480 mg BID/3 d
Intensified	120 mg TID/3 d	240 mg TID/3 d	360 mg TID/3 d

Abbreviations: BID, twice daily; TID, thrice daily.

### Pharmacokinetic and Pharmacodynamic Model

We used a published LF PK-PD model from Chotsiri et al, which was developed using PK data from children with uncomplicated malaria with and without severe acute malnutrition [12]. The lumefantrine PK model used was a 2-compartment disposition model including a 2 transit-compartments absorption model. Interindividual variability was set on the absorption rate, the relative bioavailability, and the intercompartmental rate, and an enzyme maturation effect on clearance was considered. The model included an effect of MUAC on bioavailability, where malnourished children had decreased bioavailability. The DHS survey did not collect MUAC measures, and these values were imputed by assigning children with WAZ < −2 a value between 104 and 131 mm and for children with WAZ ≥ −2 between 118 and 163 mm using a uniform distribution [12]. Ranges were reported in the Chotsiri et al study [12] and were assumed to be representative of malnourished and well-nourished populations. Additionally, the time-to-event model was implemented to assess the percentage of children remaining malaria free through 42 days.

### Population Simulations

Simulations were performed with NONMEM version 7.4.2 using the Chotsiri et al model described above to estimate LF exposures and treatment outcomes for each child. LF exposures were derived based on a child's age, weight, and MUAC values and sampled from the population parameter and variability distributions estimated by Chotsiri et al. We assumed complete adherence for each of the different dosing regimens simulated. Therefore, the results of these simulations reflect the best-case scenario.

### Target Exposure and Treatment Outcome

The target LF exposure used for children was a day 7 concentration ≥200 ng/mL as this cutoff is a predictor of 28-day malaria recurrence and 42-day recrudescence [8, 21]. Studies have reported that concentrations >200 ng/mL at day 7 are associated with >98% cure rate [15]. The simulated LF concentrations were linked to the time-to-event model to determine how many children were predicted to experience a second malaria episode.

### Analysis

Data were organized using Microsoft Excel (version 16.46) and analyzed with RStudio software version 4.1.2 (R Development Core Team). A child was defined as being underdosed if the dose they received according to their weight was less than the dose they would receive according to their expected weight.

## RESULTS

### Population Characteristics

Data from 372 363 children between 3 and 59 months of age from 25 countries with the highest percentage of malaria deaths were included. Boys and girls in every country of the DHS population had a lower median weight than the WHO child growth standard (Supplementary Figure 2). Overall, 105 376 (28%) were underweight, 140 228 (38%) were stunted, and 54 917 (15%) were wasted. Niger, India, and Chad had the highest proportions of underweight children (>30%). Most of the children were >1 year old, with 156 841 (42%) children between 1 and <3 years old, and 153 795 (41%) children between 3 and <5 years old.

### Pharmacokinetic Simulation and Dosing Regimen Optimization

#### Overall and Underweight Children Population

**Figure 1.**
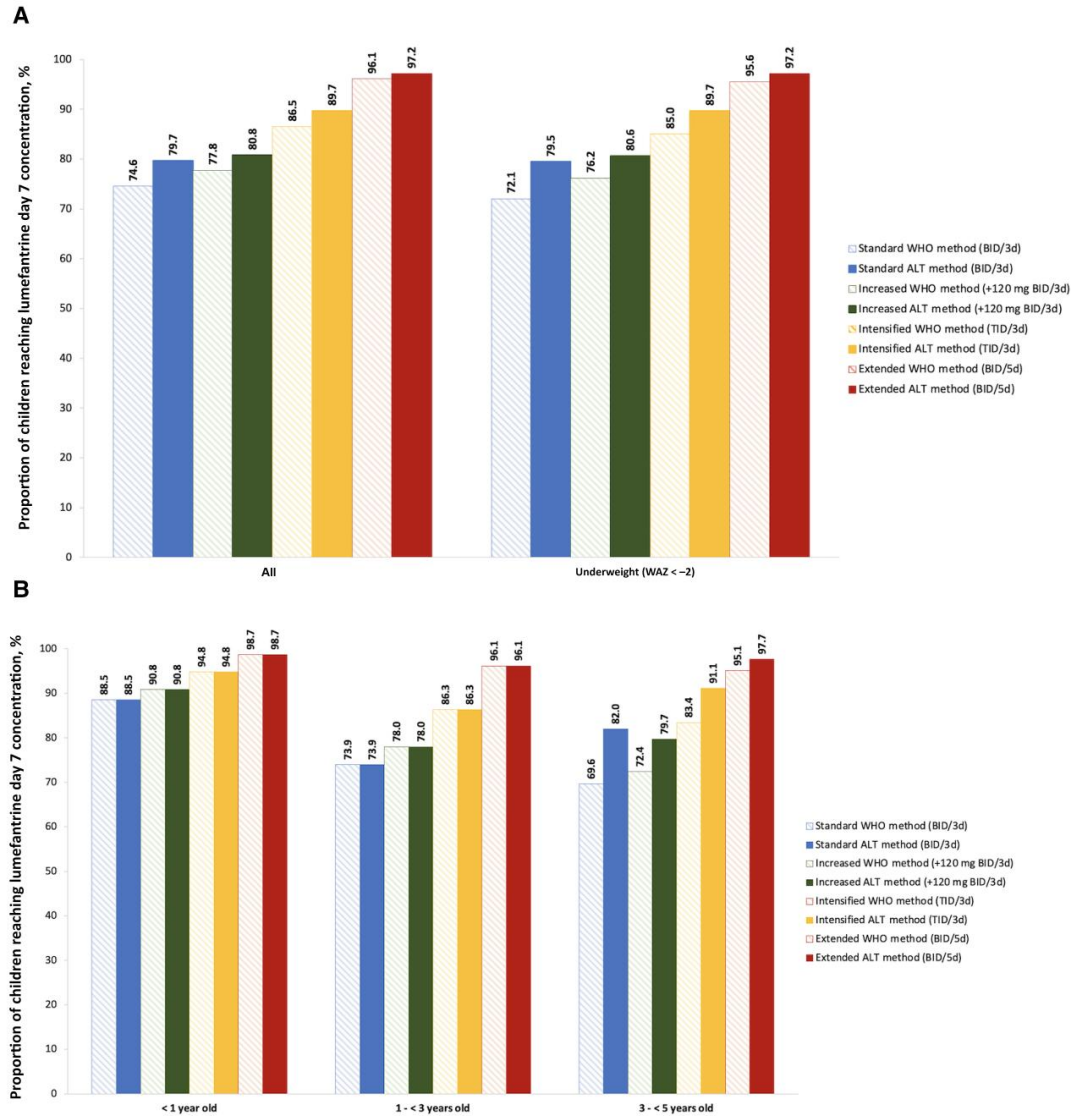
Proportion of children reaching lumefantrine day 7 target concentration of 200 ng/mL, with the 4 tested dosing regimens, based on World Health Organization and alternative methods, overall and in underweight (weight-for-age *z*-score < −2) children (*A*) and in each age band (*B*). Abbreviations: ALT, alternative; BID, twice daily; TID, thrice daily; WAZ, weight-for-age *z*-score; WHO, World Health Organization.

In the overall (WAZ < −2 and WAZ ≥ −2) and underweight (WAZ < −2) child population, we modeled that the extended dosing regimen allowed the highest percentage of children reaching the LF day 7 concentration threshold of 200 ng/mL, following by the intensified dosing regimen, while the increased and the standard dosing regimen led to the lowest rate (Figure 1*A*). With the standard LF regimen, children were less likely to reach the target LF concentration of 200 ng/mL as compared to all 3 adjusted regimens (WHO method: overall population: 74.6%, underweight population: 72.1%; ALT method: overall population: 79.7%, underweight population: 79.5%). For the extended regimen, children were the most likely to reach the target concentration, in the overall and underweight populations (WHO method: overall population: 96.1%, underweight population: 95.6%; ALT method: overall population: 97.2%, underweight population: 97.2%). The extended regimen was estimated to increase the percentage of children who reached the target LF concentration by 21.5% in the overall population with WHO method dosing and 17.5% with ALT method dosing, and by 23.5% in the underweight population with WHO method dosing and 17.7% with ALT method dosing. The increased adjusted regimen (120 mg LF added to each dose) was not predicted to improve LF concentrations compared with the standard regimen (WHO method: overall population: 77.8%, underweight population: 76.2%; ALT method: overall population: 80.8%, underweight population: 80.6%). For standard and adjusted regimens, a higher percentage of children were modeled to reach target LF concentrations with ALT method dosing compared to WHO method dosing, with larger percentage gains made in the underweight population (difference of percentage between WHO and ALT methods in overall population—standard: 5.1%, increased: 3%, intensified: 3.2%, extended: 1.1%; in underweight population—standard: 7.4%, increased: 4.4%, intensified: 4.7%, extended: 1.6%) (Figure 1*A*).

#### Exploration of Age-Band Dosing

We only observed a benefit of dosing either by age and extending/intensifying in children >3 years old, with an increase of proportion of children meeting the target concentration (WHO method: standard: 69.6%, increased: 72.4%, intensified: 83.4%, extended: 95.1%; ALT method: standard: 82.0%, increased: 79.7%, intensified: 91.1%, extended: 97.7%). The extended dosing regimen provided the best drug efficacy, with 16% more children reaching the exposure target compared to the standard dosing regimen (Figure 1*B*). This observation is also made at the country level (Supplementary Figure 3).

### Pharmacodynamic Simulations

**Figure 2.**
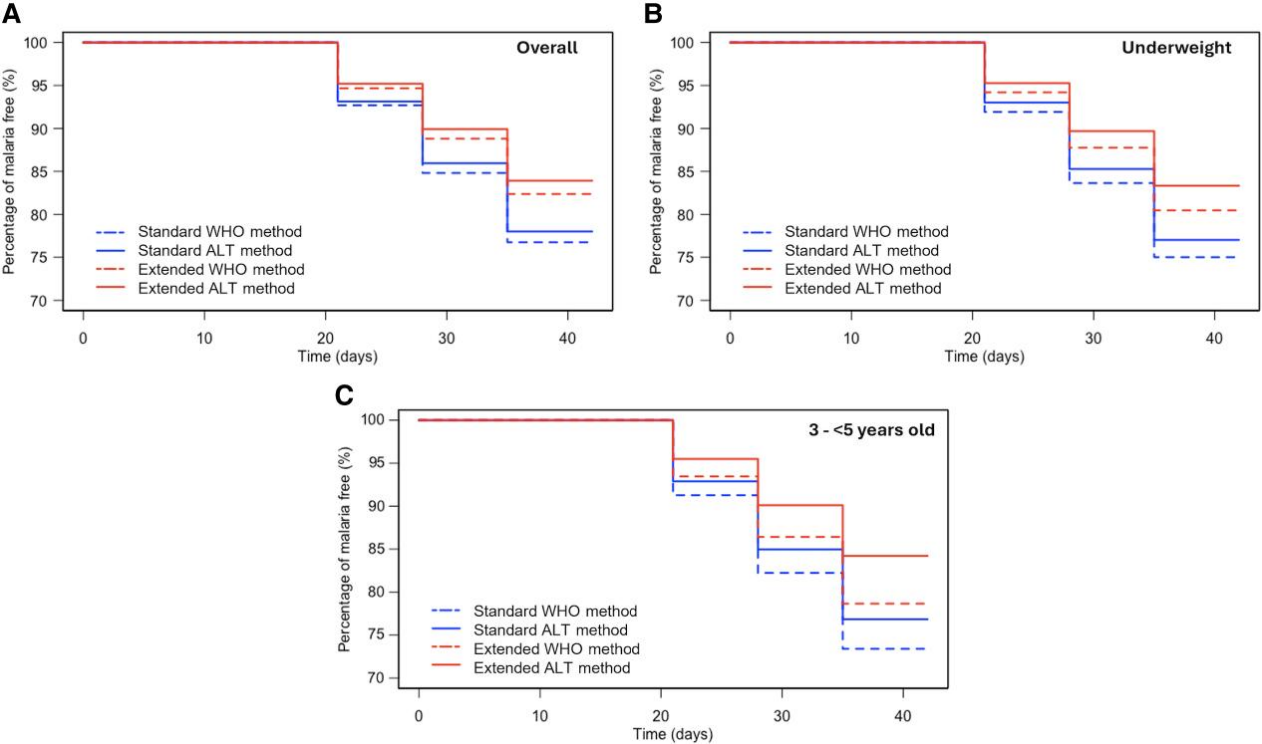
Percentage of malaria-free children overall (*A*), among underweight children (*B*), and in the age band 3–5 years (*C*) in the Demographic and Health Surveys population over 42 days of observation, based on World Health Organization (WHO) and alternative (ALT) methods, and for the less and the more suitable dosing regimen, respectively, standard and extended.

Simulation results by nutritional status and age can be found in Supplementary Figure 4 and Supplementary Table 1. Focusing on the endpoint of no reinfection for 42 days, we observed that extended dosing regimen provided a higher percentage of children being malaria free over 42 days (WHO method: 82%; ALT method: 84.1%) than the standard dosing regimen (WHO method: 76.7%; ALT method: 78%) (Figure 2*A*). The same relationship was observed in the underweight group (WHO method: standard: 75.0%, extended: 80.4%; ALT method: standard: 76.8%, extended: 83.2%) (Figure 2*B*) and the 3- to 5-year-old populations (WHO method: standard: 73.5%, extended: 78.6%; ALT method: standard: 76.9%, extended: 83.9%) (Figure 2*C*) (Supplementary Table 1).

## DISCUSSION

Using real-world anthropometric data from nationally representative DHS surveys, we simulated LF exposure for 372 363 young children using a model developed by Chotsiri et al, which quantified the impact of malnutrition on LF PK. We calculated the percentage of children with a LF concentration >200 ng/mL after 7 days of treatment, and the proportion of children without reinfection up to 42 days. We show that although 74% of children would have reached target LF concentrations with current WHO-recommended standard regimens, extending dosing of the LF treatment course to 5 days and using expected body weight to dose malnourished individuals (ALT method) would be needed to achieve target LF concentrations for >95% of children under 5 years of age. We showed that extending the LF treatment course to 5 days was predicted both to minimize differences in LF day 7 concentrations by nutritional status, even if alternative dosing for expected body weight could not be implemented for malnourished children, and it would increase the percentage of individuals who achieve target concentrations in the whole population. Using thrice-daily dosing of LF was also predicted to improve LF exposure to a lesser degree. In the setting of potential decreased sensitivity to artemether and LF in high-burden countries [22], the WHO-recommended dosing should be reviewed to contribute in malaria eradication strategy optimization.

WHO recognizes that LF exposure is suboptimal in children <3 years old [3]. We found that children between 3 and 59 months of age would be impacted by suboptimal LF exposure, with the 36- to 59-month age group having the lowest LF levels, regardless of nutritional status. When compared to adults, the recommended WHO method LF dosing regimen results in 24% and 13% lower median day 7 concentration in children weighing <15 kg and 15–24 kg, respectively [23]. It is crucial to optimize drug exposure to cure but also to prevent malaria reinfection [24]. Because of absorption saturation, increased doses of LF are inefficient to increase exposure; we showed no improvement in the proportion of children predicted to achieve the desired efficacy targets when 120 mg of LF was added to the recommended dosing regimen. This results support that LF saturation is observed and modeled as a 43% decrease in bioavailability by Chotsiri et al [12]. However, we showed that LF exposure can be increased by extending treatment to 5 days instead of the recommended 3 days, and by intensifying it with a dose given thrice daily instead of twice daily. Those results are consistent with the results of Chotsiri et al and a pooled analysis by the WorldWide Antimalarial Resistance Network LF PK/PD Study Group [12, 15]. Another study from Whalen et al showed that LF exposure was increased in young children with an AUC cumulative 1.82-fold higher with a 5-day regimen, as well as the median LF concentrations at days 7, 14, and 21, compared to the standard dosing regimen [20]. LF is safe and well tolerated without serious adverse events, making it reasonable to extend treatment from a safety perspective [15].

We showed that the ALT method dosing increased the percentage of malnourished children who reached the LF concentration threshold associated with treatment success. Understanding why young children are prone to lower drug exposures, and the underlying mechanism of this, is essential for dose adjustment. Considering that LF PK characteristics evolve with child growth and enzymatic maturation during the first years of life, expected-weight-based dosing could be relevant. Indeed, the LF is metabolized by cytochrome P450 3A4 (CYP3A4) and is a substrate of P-glycoprotein, which is immature at birth, limiting oral bioavailability [20, 25]. To this respect, studies showed lower LF exposures in young children compared to adults [10]. Studies have also shown that severe malnutrition decreases LF bioavailability significantly [12, 18]. Chotsiri et al investigated malnutrition indicators’ (MUAC, WHZ, and WAZ) effect on LF PK parameters, and concluded that they had a significant impact on the relative bioavailability [9]. This could result from pathophysiological changes that malnutrition can cause in malnourished children as a decrease or a delay in absorption, higher body water level, or reduced albumin concentration [26]. A pooled analysis also showed that with decreasing WAZ score, children have higher risk of recrudescent malaria and lower day 7 concentration [15].

Other physiological factors can impact pharmacokinetics and counteract those expectations, as presented by Kloprogge et al [23]. In this analysis, young children in the 10–15 kg weight band are at a higher risk of receiving a lower mg/kg total dose of AL, reducing predicted drug efficacy [27]. WHO growth charts consider that child anthropometric factors are closely linked. Thus, dosing based on weight, age, or height should result in the same dosing [19]. However, multiple factors can modify these relationships, such as malnutrition, where the anthropometric balance is compromised. To bear our assumption that the dosing regimen should incorporate expected body weight, which is based on age, instead of actual body weight alone, we stratified our results by age bands of <12, 12 to <36, and 36–59 months. We only identified the 36- to 59-month-old, malnourished children as being disproportionately impacted by currently recommended weight-based dosing. This result can be explained by the fact that children in the age band 36–59 months straddle between 2 LF dosing levels because of their varying weight around 15 kg. Thus, although malnourished children would be expected to have similar maturation and quantities of metabolizing enzymes compared with their better-nourished peers, they would be more likely to fall within the lower weight band and receive lower LF doses. Additional studies are needed to determine if revised weight bands could minimize this problem, as done for piperaquine [28].

In addition to malnutrition being prevalent and a risk for low LF exposure, studies have shown that chronic malnutrition in young children is associated with an increased risk of malaria infection [18, 29]. Thereby, malnourished children might need more drug exposure to achieve similar outcomes and suboptimal drug concentrations could select for drug resistance, a growing concern for LF in some parts of Africa [30]. However, drug administration in young children can be difficult, and to increase the tablets’ intake might be challenging. Indeed, without existing adapted formulation, tablets are crushed and mixed with food or water to facilitate ingestion. Doing so, the bitter taste of tablets is released and may lead children to spit them out, resulting in in suboptimal dosing. Some pediatric formulations exist, such as syrups, but their conservation and accurate dosing is uncertain. The advantage in using artemether-lumefantrine in young children is the availability of Coartem Dispersible tablets, especially developed for young children, that solve taste and storage issues [2, 31].

This study has some limitations. First, the Chotsiri et al model had the MUAC parameter as a covariate on bioavailability, whereas MUAC data were not collected in the DHS program. We selected a conservative approach to not overestimate the malnutrition burden and attributed median values from the Chotsiri et al study. This assumption may introduce bias when calculating the malnutrition prevalence. Second, we assumed a total adherence to LF treatment, and simulation results reflect an ideal scenario. Patient compliance depends on the number of doses and timing [32]. Those information are complex to access while they are crucial to ensure treatment success and to limit recurrence and recrudescence of malaria [14, 33]. Finally, the data were collected from the year 1994 to 2018 according to country. Many efforts have been undertaken in the last years to control malnutrition, and evolving environmental factors suggest that data have most likely changed over 20 years. The availability and consistency of recent data would increase the robustness of our study results.

In conclusion, malaria remains a leading cause of mortality and morbidity in young children globally. Using real-world data and PK-PD modeling, we quantified the impact of underdosing of LF in young children on malaria treatment outcomes in countries with the highest malaria burden [12, 15] and on a larger global scale than clinical trials can make. We showed that extending by 2 extra days of twice-daily dosing and adjusting dosing by expected weight for malnourished children could together significantly increase the proportion of children reaching the LF day 7 concentration threshold of 200 mg/L by 22.6%. This represents 84 154 children, of whom 26 680 (31.7%) are malnourished and who could achieve LF concentrations associated with improved treatment outcomes. These findings support further investigation of the 5-day LF treatment regimen and consideration of age and weight when dosing LF for uncomplicated malaria treatment globally.

## Supplementary Material

ofae627_Supplementary_Data

## Supplementary Data

Supplementary materials are available at *Open Forum Infectious Diseases* online. Consisting of data provided by the authors to benefit the reader, the posted materials are not copyedited and are the sole responsibility of the authors, so questions or comments should be addressed to the corresponding author.

## Notes

***Author contributions.*** E. H., R. M. S., S. S., and E. W. contributed to conceptualization and methodology. B. P. S., E. H., and R. M. S. contributed to supervision. B. P. S., E. W., and R. M. S. contributed to writing–review and editing. S. S. contributed to investigation, methodology, writing–original draft, visualization, and formal analysis. R. M. S. contributed to validation. All authors critically revised the article and approved the final version for publication.

***Ethics approval.*** No ethics committee approval was required for this study, and this study does not include factors necessitating patient consent.
