# Supplementary material for: Optimizing Lumefantrine Dosing for Young Children in High-Malaria-Burden Countries Using Pharmacokinetic-Pharmacodynamic Simulations
Source: Open Forum Infect Dis. 2024 Oct 17;11(11):ofae627. doi: 10.1093/ofid/ofae627 (PMC11561580; doi:10.1093/ofid/ofae627)
Supplement: ofae627_Supplementary_Data [file ofae627_supplementary_data.docx]

Table S1. Percentage of children malaria-free over 42 days of observation in each age band, with the four tested dosing regimen, based on WHO- and ALT-method.

|  |  |  | **Malaria-free children (%)** | |
| --- | --- | --- | --- | --- |
|  |  | **Time (days)** | **28** | **42** |
| **All children** | **WHO-method** | **Standard** | 84.7 | 76.7 |
|  |  | **Extended** | 88.7 | 82.2 |
|  |  | **Increased** | 85.5 | 77.6 |
|  |  | **Intensified** | 87.8 | 81.0 |
|  | **ALT-method** | **Standard** | 85.6 | 78.0 |
|  |  | **Extended** | 89.8 | 84.1 |
|  |  | **Increased** | 85.9 | 78.2 |
|  |  | **Intensified** | 89.0 | 82.5 |
| **Underweight** | **WHO-method** | **Standard** | 83.7 | 75.0 |
|  |  | **Extended** | 87.6 | 80.4 |
|  |  | **Increased** | 84.3 | 75.9 |
|  |  | **Intensified** | 86.4 | 78.9 |
|  | **ALT-method** | **Standard** | 85.0 | 76.8 |
|  |  | **Extended** | 89.6 | 83.2 |
|  |  | **Increased** | 85.1 | 76.8 |
|  |  | **Intensified** | 88.6 | 81.7 |
| **< 1 year** | **WHO-method** | **Standard** | 88.2 | 81.2 |
|  |  | **Extended** | 92.9 | 88.3 |
|  |  | **Increased** | 89.4 | 83.1 |
|  |  | **Intensified** | 91.5 | 86.3 |
|  | **ALT-method** | **Standard** | 88.3 | 81.2 |
|  |  | **Extended** | 92.8 | 88.1 |
|  |  | **Increased** | 89.1 | 82.7 |
|  |  | **Intensified** | 91.8 | 86.8 |
| **1-<3 years** | **WHO-method** | **Standard** | 83.0 | 74.1 |
|  |  | **Extended** | 88.0 | 80.4 |
|  |  | **Increased** | 84.0 | 75.1 |
|  |  | **Intensified** | 86.6 | 78.5 |
|  | **ALT-method** | **Standard** | 83.2 | 74.2 |
|  |  | **Extended** | 88.2 | 80.7 |
|  |  | **Increased** | 84.2 | 75.4 |
|  |  | **Intensified** | 86.7 | 78.8 |
| **3-<5 years** | **WHO-method** | **Standard** | 82.2 | 73.5 |
|  |  | **Extended** | 86.3 | 78.6 |
|  |  | **Increased** | 82.4 | 73.4 |
|  |  | **Intensified** | 85.1 | 76.9 |
|  | **ALT-method** | **Standard** | 85.1 | 76.9 |
|  |  | **Extended** | 90.1 | 83.9 |
|  |  | **Increased** | 83.9 | 75.4 |
|  |  | **Intensified** | 88.9 | 82.2 |

**(a)**
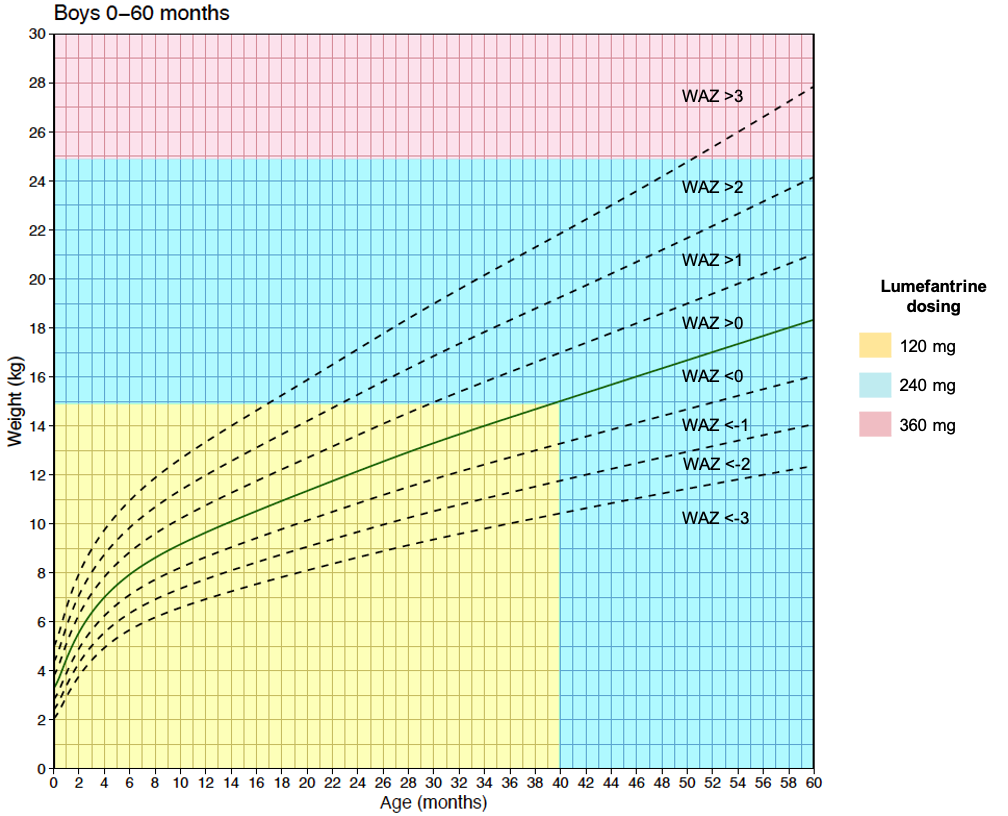


**(b)**
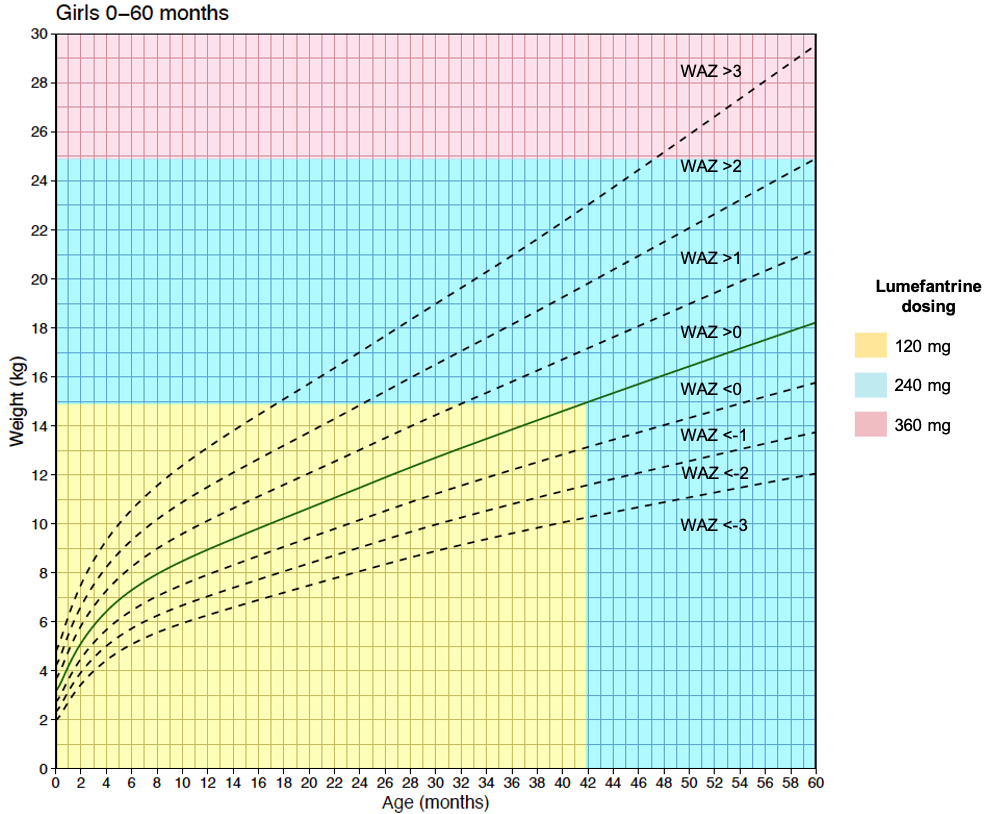


Figure S1. Proposed dosing chart for (a) boys, and (b) girls 0-60 months. WAZ = weight-for-age z score.

**(a)**


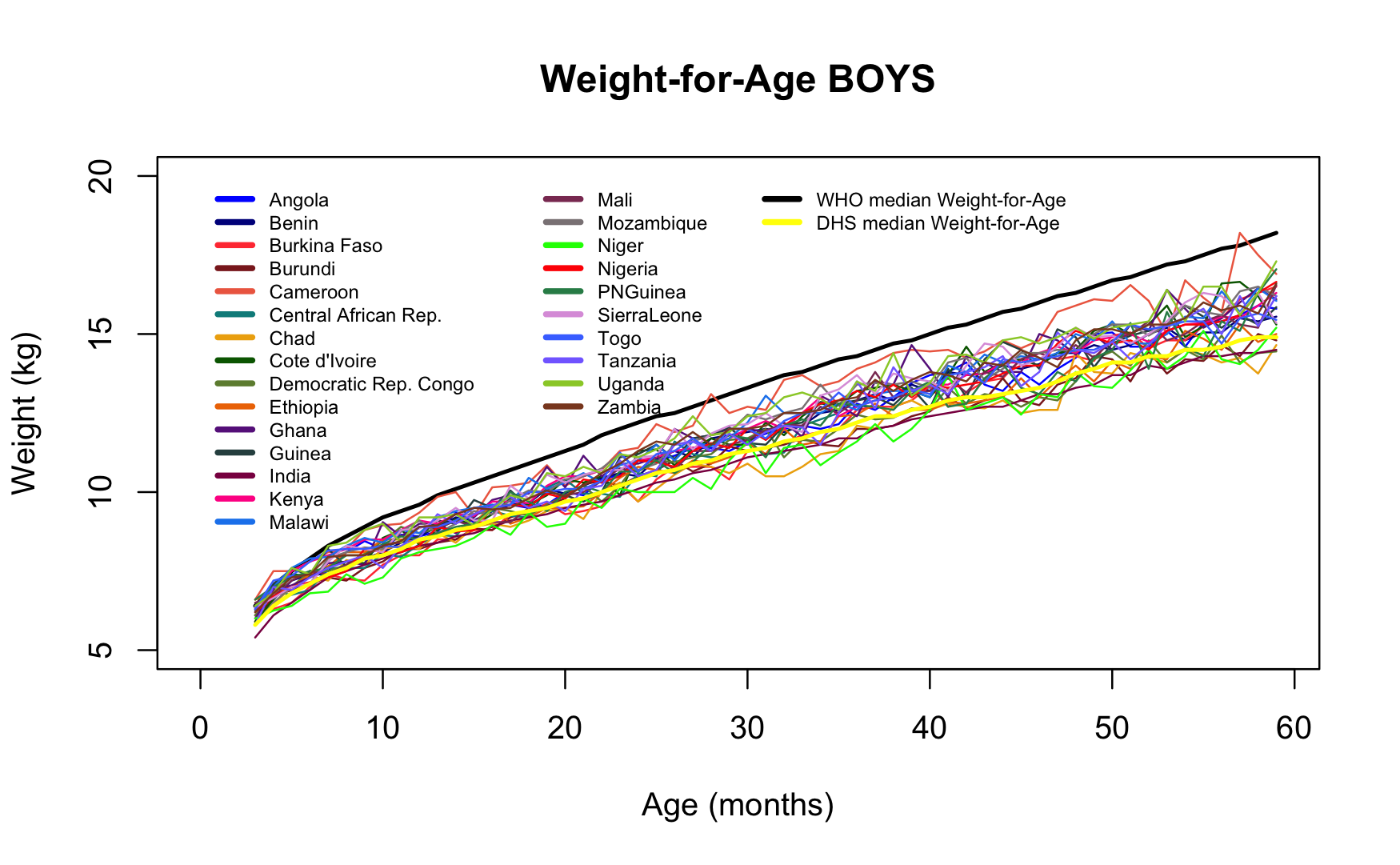


**(b)**


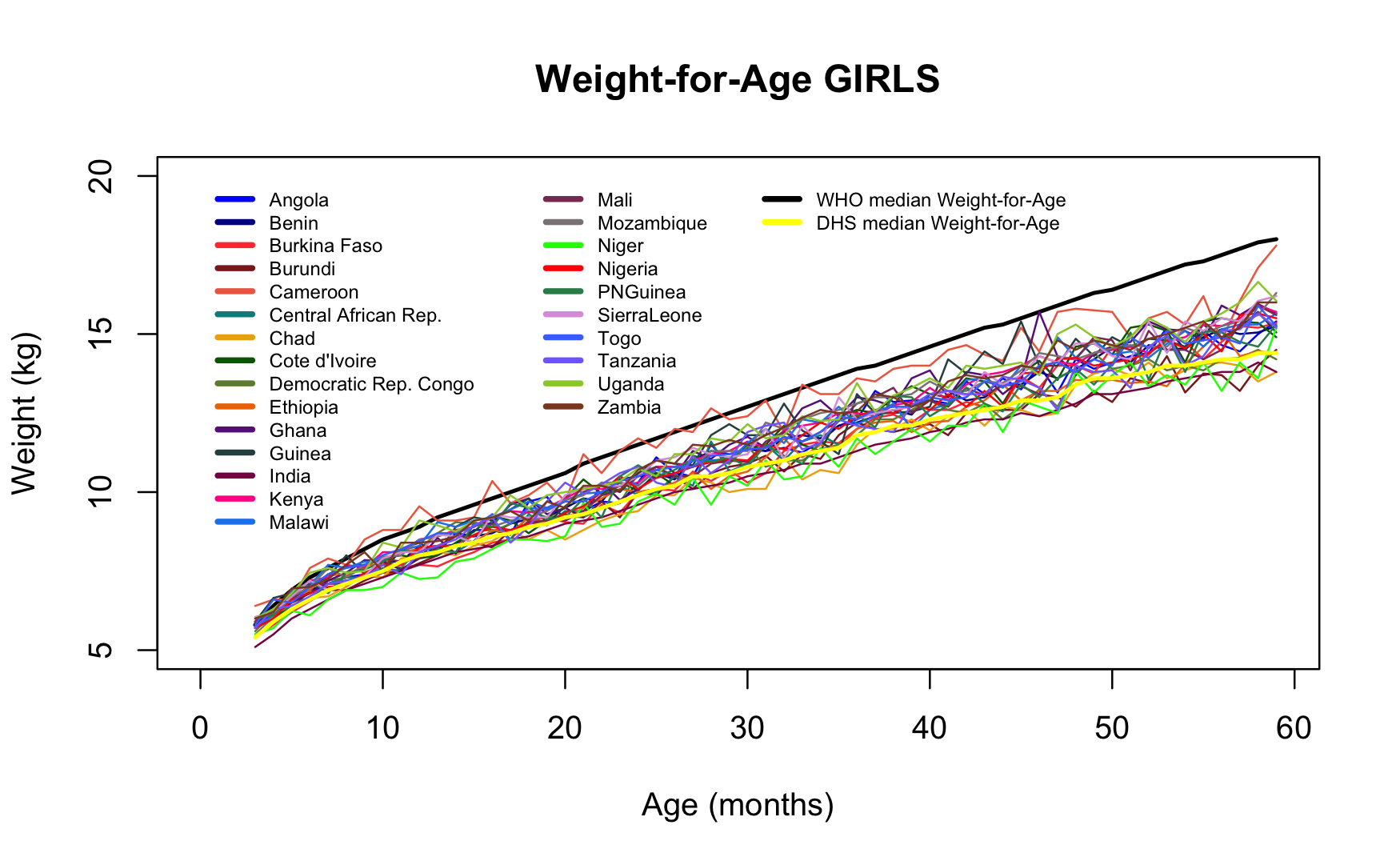
Figure S2. (a) Boys and (b) girls median weight as a function of the age for each country. The black line represents the median weight trajectory based on the WHO growth standards, and the yellow line is the median of the DHS population.


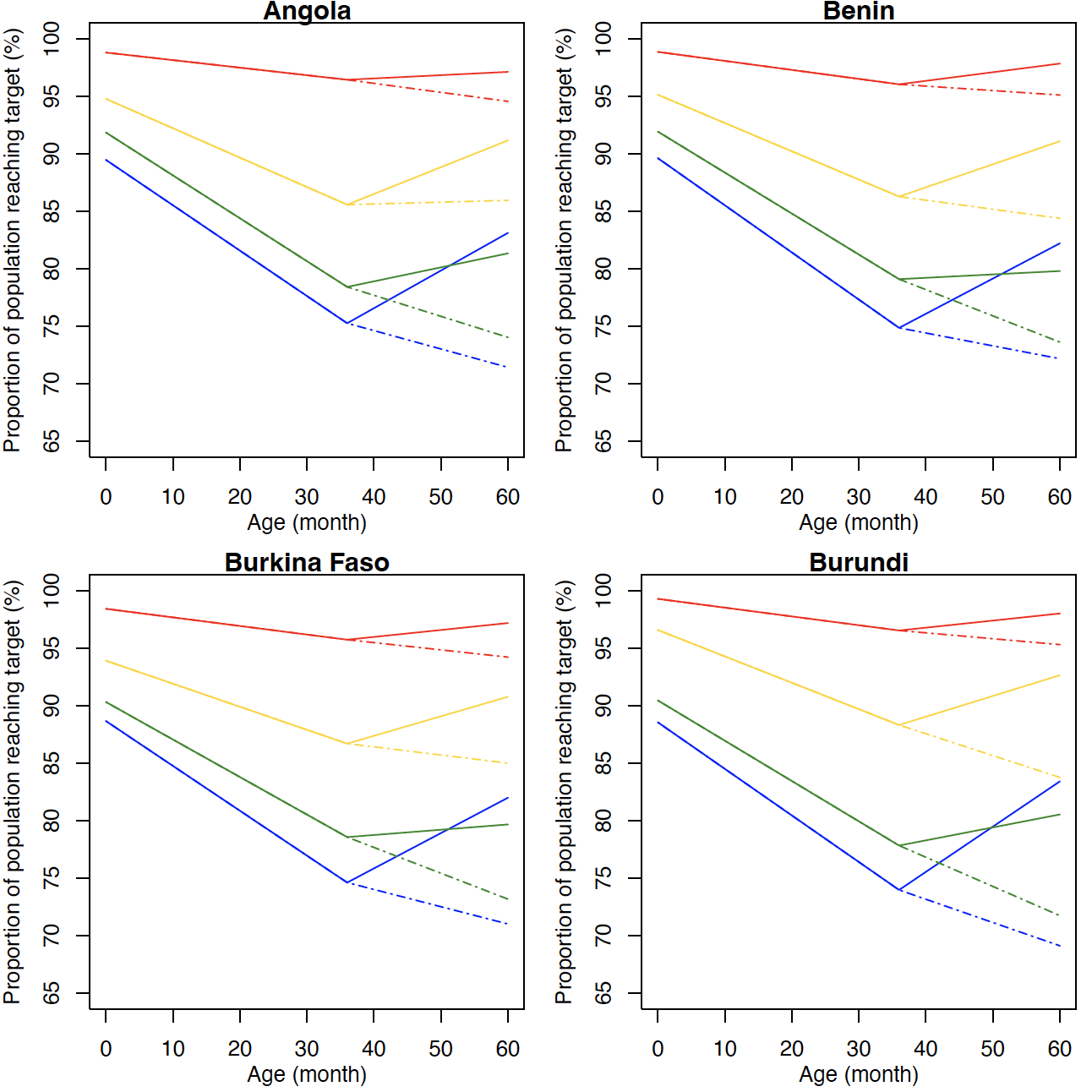


Figure S3. Proportion of DHS children in each country with a lumefantrine day-7 concentration over 200 ng/mL, with the four tested dosing regimens, using WHO- (dashed lines) and ALT-method dosing (solid lines).


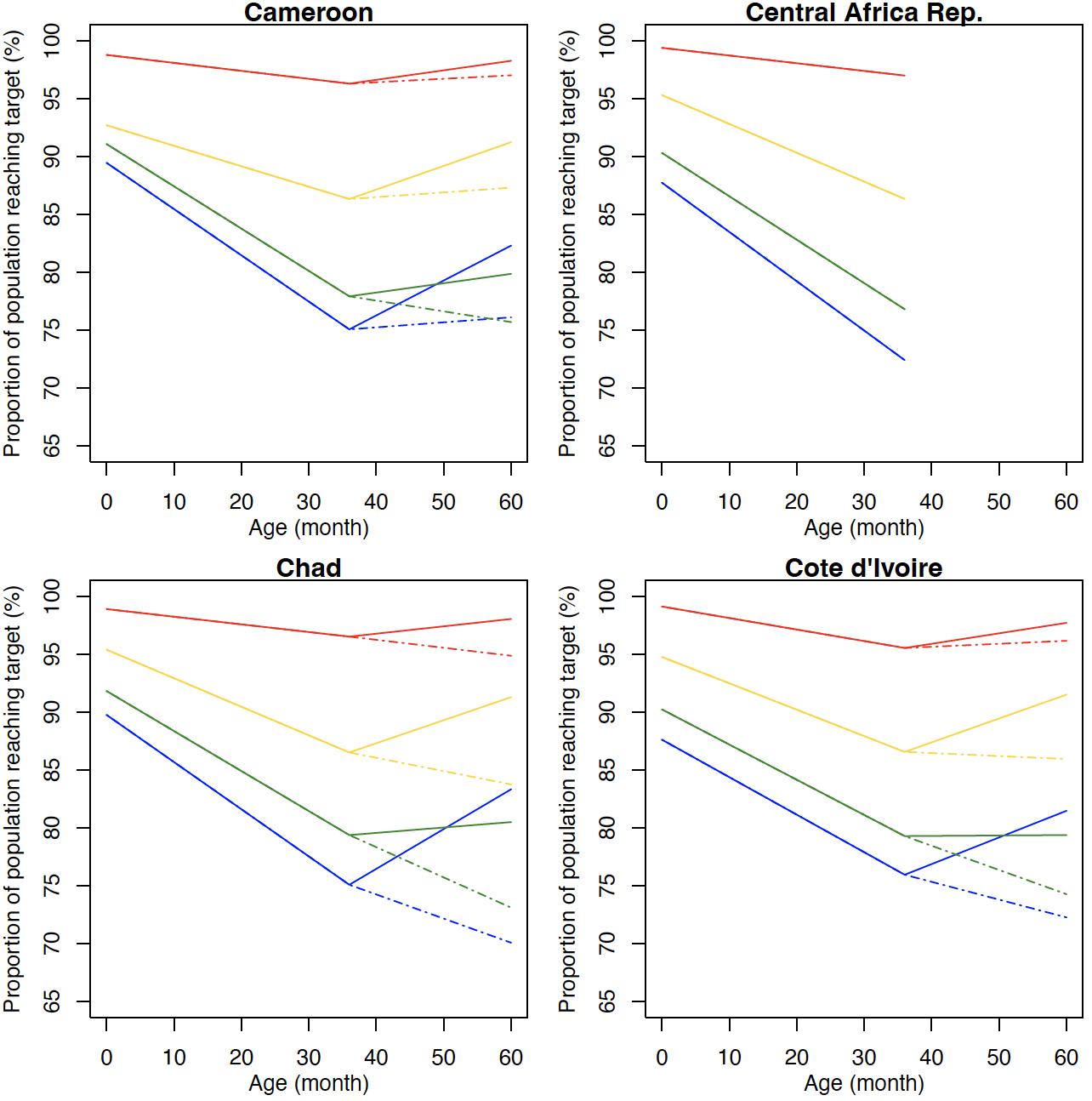


**(Figure S3. To be continued)**


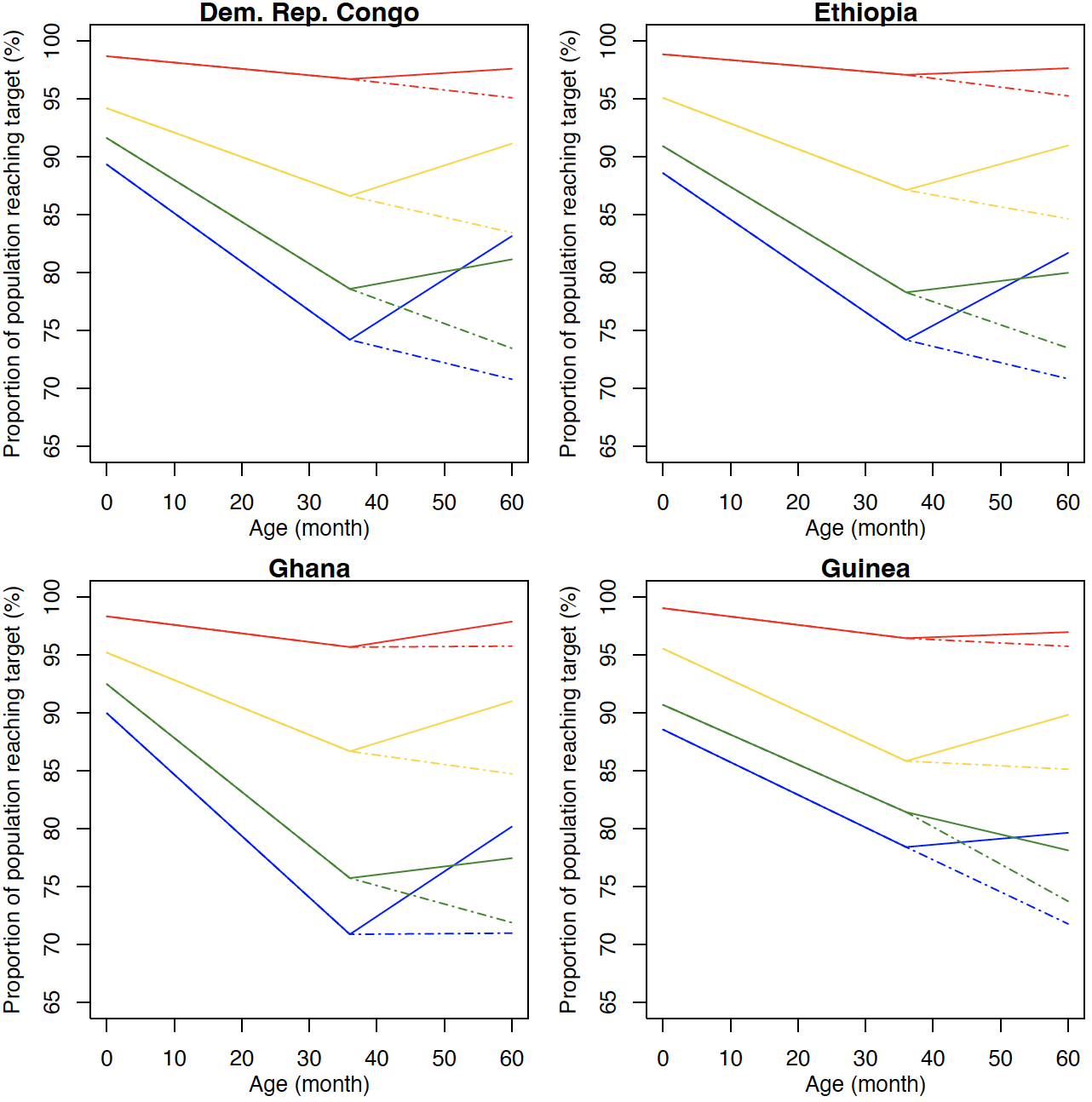


**(Figure S3. To be continued)**


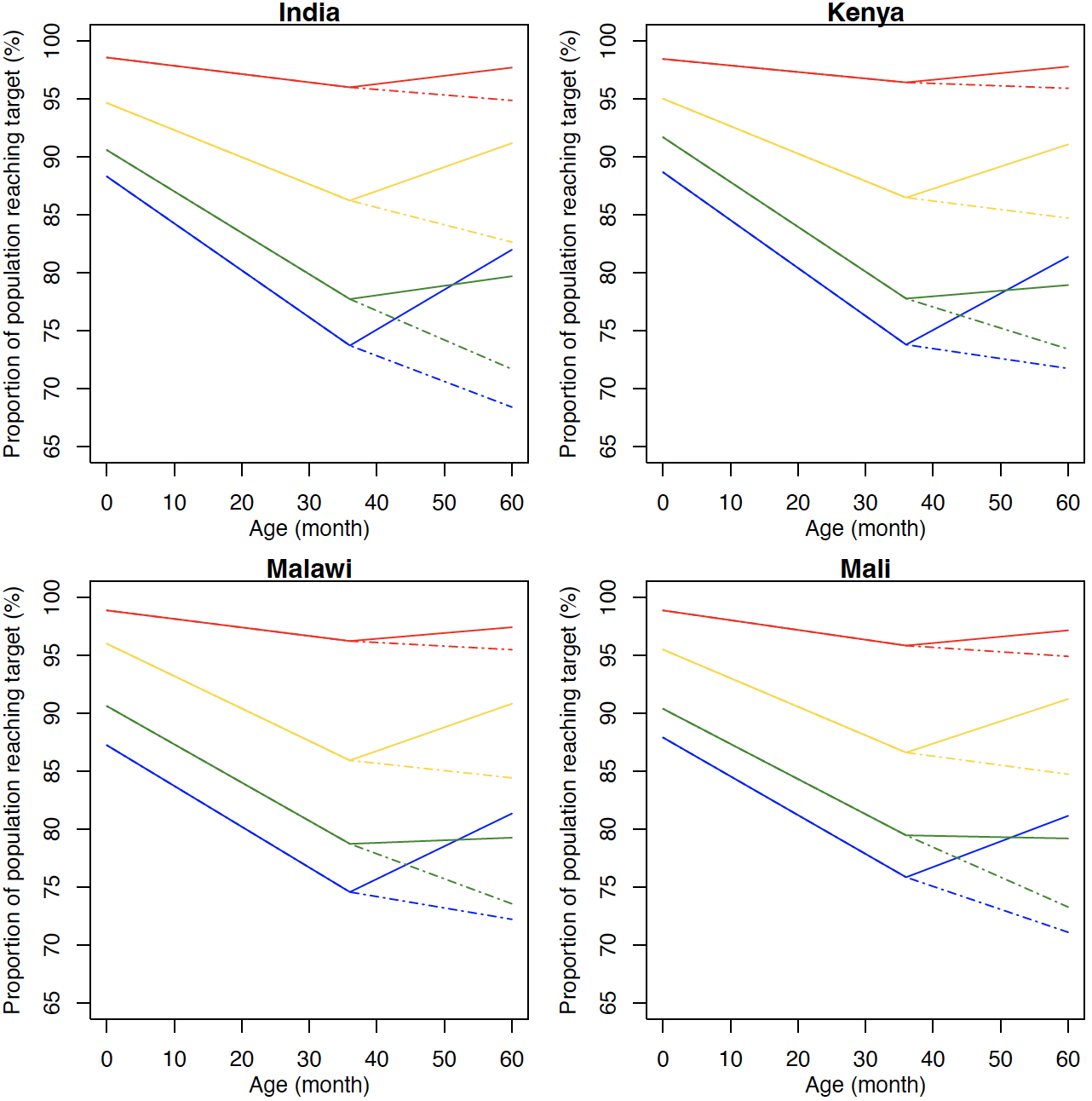
**(Figure S3. To be continued)**


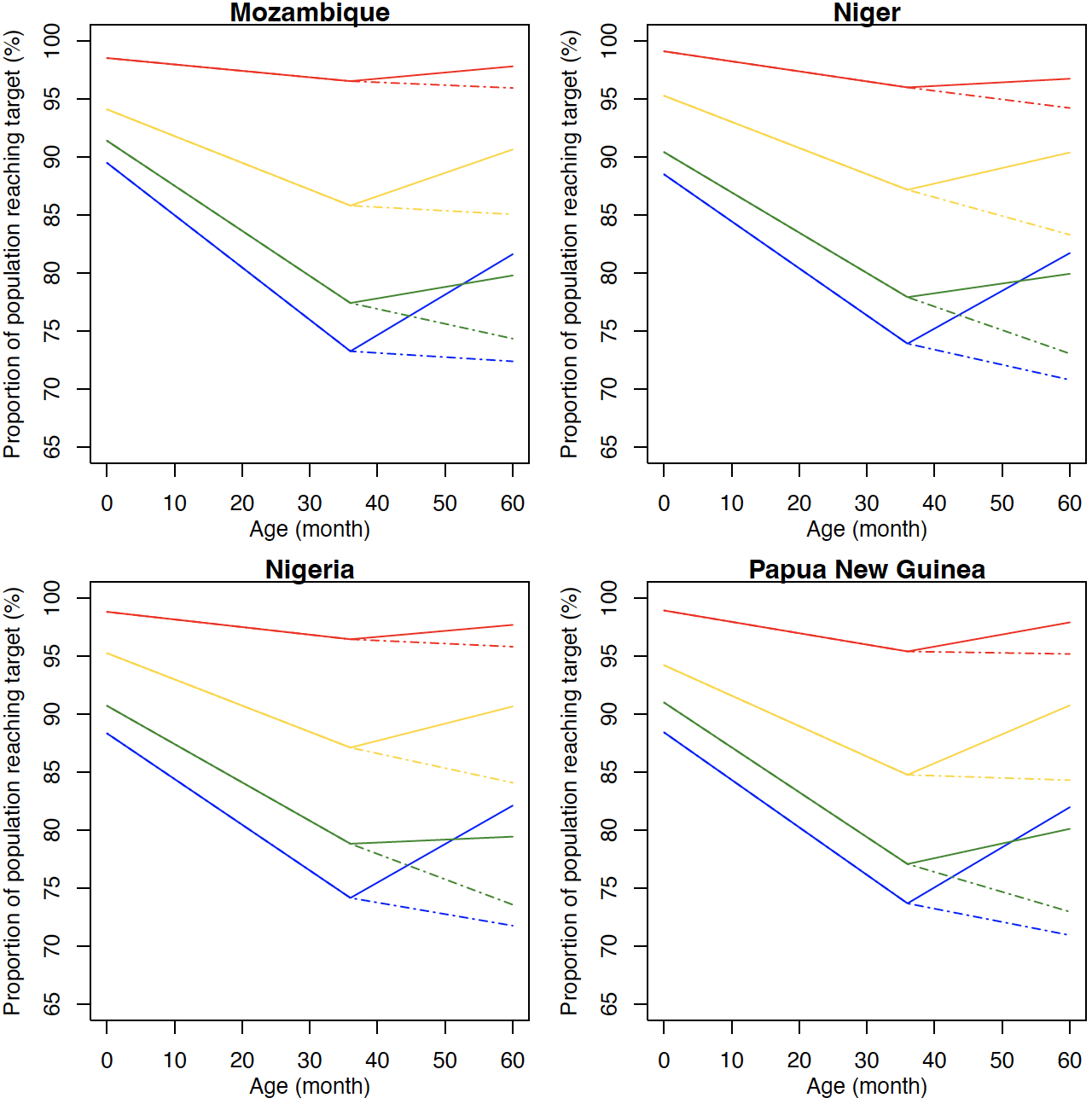
**(Figure S3. To be continued)**


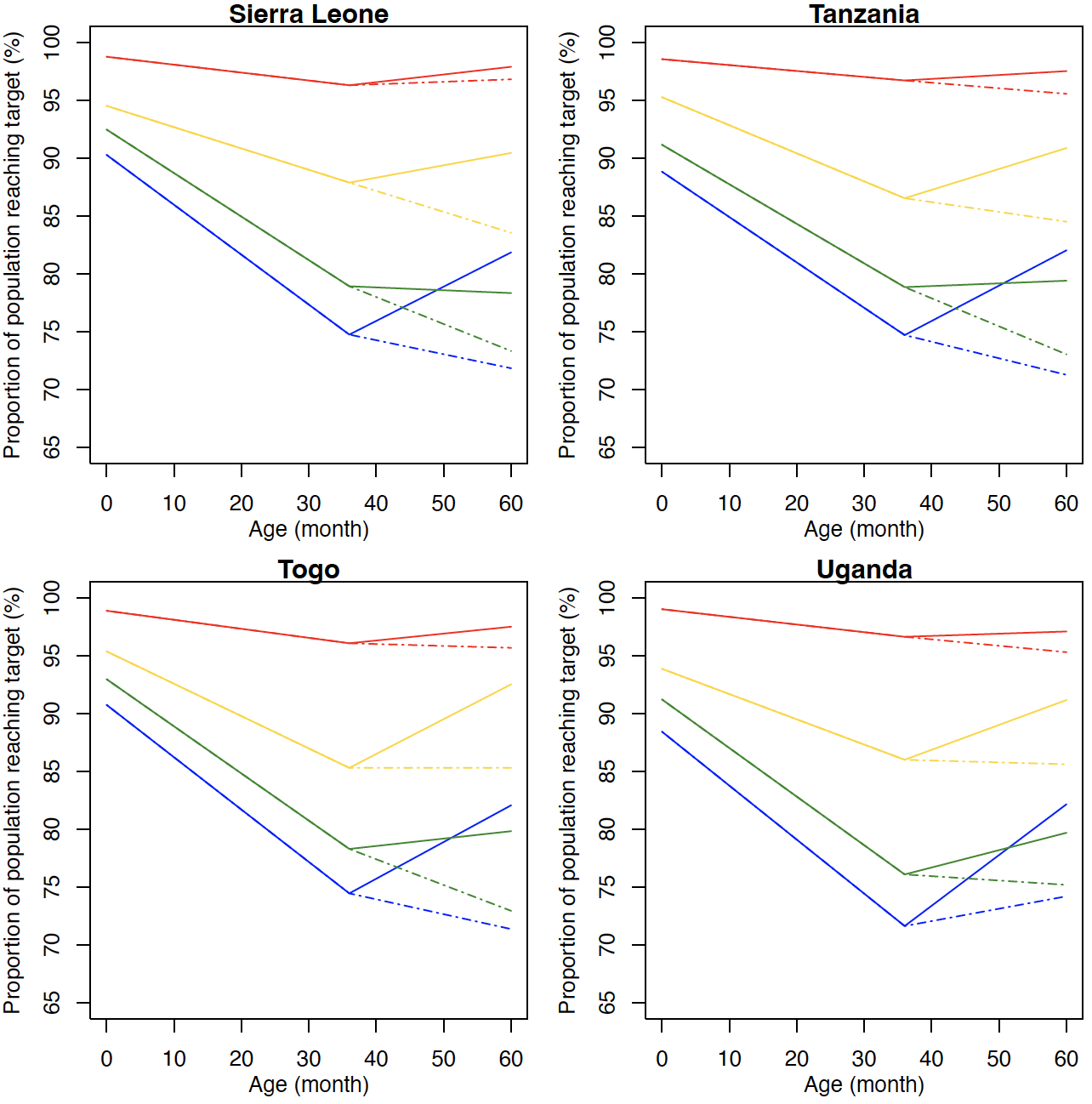
**(Figure S3. To be continued)**


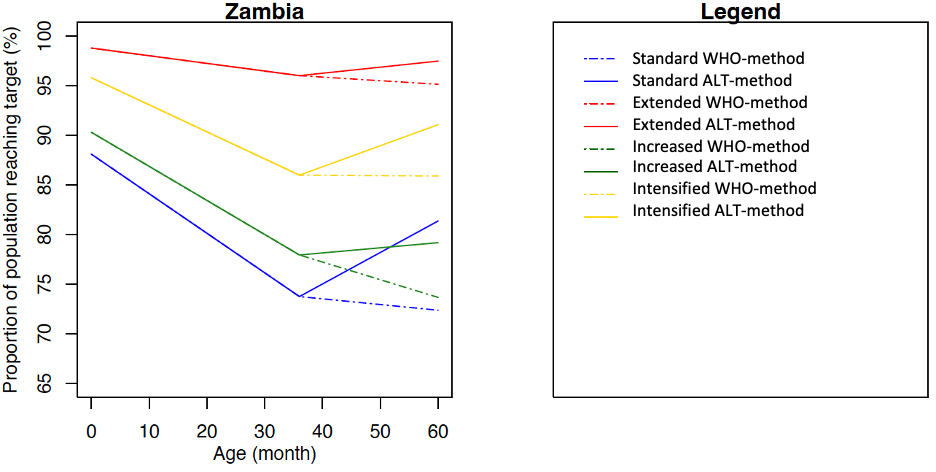


**(Figure S3. End)**

**(a)**
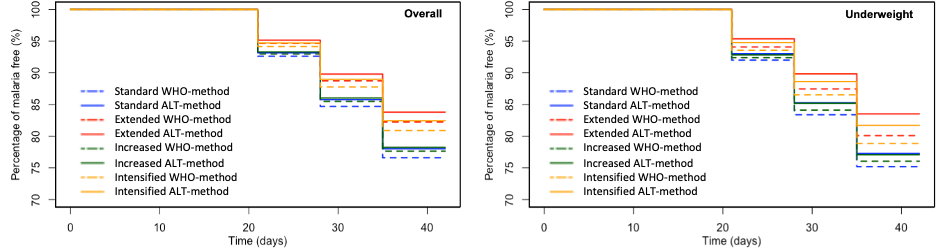


(b)
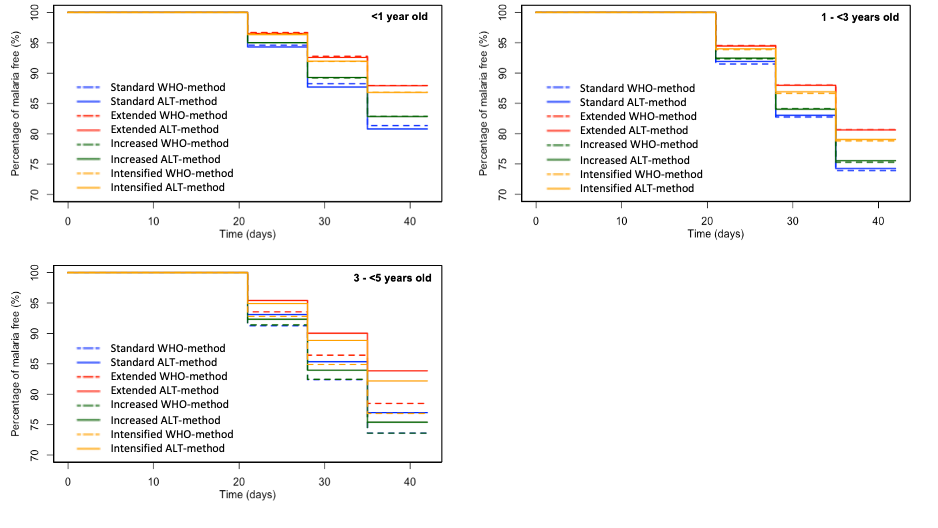
Figure S4. Percentage of children malaria-free in: (a) the overall (left) and the underweight (right) DHS population, (b) stratified by age, and for the four tested dosing regimen, based on WHO- and ALT-method.
